# Unsaturated Poly(Hydroxyalkanoates) for the Production of Nanoparticles and the Effect of Cross-Linking on Nanoparticle Features

**DOI:** 10.3390/ma12060868

**Published:** 2019-03-15

**Authors:** Rosario Pignatello, Giuseppe Impallomeni, Sarha Cupri, Giuseppe Puzzo, Claudia Curcio, Maria Giovanna Rizzo, Salvatore Guglielmino, Alberto Ballistreri

**Affiliations:** 1 Department of Drug Sciences, Section of Pharmaceutical Technology; University of Catania, Catania 95125, Italy; saracupri@yahoo.it (S.C.); giuseppepuzzo3@virgilio.it (G.P.); clacurcio@virgilio.it (C.C.); 2 NANO-I, Research Center on Ocular Nanotechnology, University of Catania, Catania 95125, Italy; aballistreri@unict.it; 3 CNR, Institute for Polymers, Composites and Biomaterials, Catania 95126, Italy; gimpa@unict.it; 4 Department of Chemical, Biological, Pharmaceutical and Environmental Science, University of Messina, Messina 98166, Italy; mgrizzo@unime.it (M.G.R.); sgugliem@unime.it (S.G.); 5 Department of Drug Sciences, Section of Chemistry; University of Catania, Catania 95125, Italy

**Keywords:** polymers, polyesters, calcein, Nile red, freeze-drying, cryoprotectants

## Abstract

A biodegradable poly(3-R-hydroxyalkanoate) synthesized by *Pseudomonas mediterranea* was investigated as a biomaterial to obtain colloidal drug delivery systems. Using a nanoprecipitation method, nanoparticles with a mean size of 155 nm and a negative surface charge were formed. They can be freeze-dried by adding hydroxypropyl-β-cyclodextrin as a cryoprotectant, and they have been shown to efficiently load both a hydrophilic (calcein) and a lipophilic (Nile red) model probe. Since this polymer contains terminal double bonds in the side chains, cross-linking conditions were tested. In particular, under the action of UV rays or irradiation with an incandescent yellow lamp, this polymer tended to cross-link.

## 1. Introduction

Drug delivery systems (DDS) have been developed to carry the pharmacologically active principle to the target organ or district with the highest specificity possible, and with an (ideally) programmed kinetic of release, therefore reducing the amount of drug that is administered to the system and adverse collateral effects. To produce DDS, it is highly desirable to use non-toxic and biocompatible polymers [1,2,3]. Among them, poly(hydroxyalkanoates) (PHA) are macromolecules that have been investigated in the last years as biodegradable and biocompatible biomaterials, and as matrices for the production of micro- and nano-sized drug carriers [4,5,6,7].

**Figure 1:**
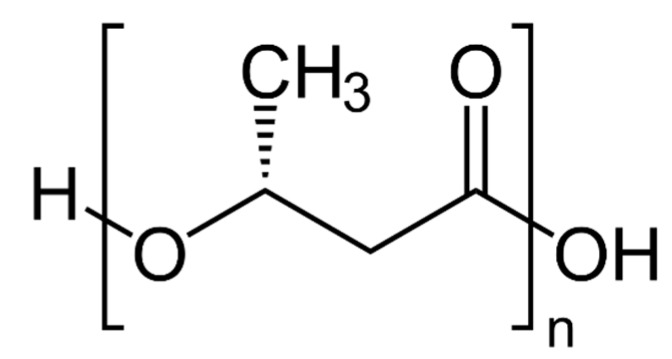
General structure of poly(3-R-hydroxyalkanoates) (PHA).

PHA are synthesized by more than 90 Gram-positive and Gram-negative bacterial genera [8] in cultures lacking some nutrient (for instance, nitrogen), and are accumulated in the bacterial cell for energy storage as granules constituting up to 90% of the cell dry weight [9]. Their structure is shown in Figure 1.

The main group of PHA is that of poly-(3-R-hydroxyalkanoates), which are stereoregular polymers. Depending on the R side chain length, these polymers are classified as short-chain length (scl)-PHA (R is a methyl or an ethyl group) and medium-chain length (mcl)-PHA (R from propyl to tridecyl group). Bacteria of the *Ralstonia* genus produce scl-PHA; those of the *Pseudomonas* genus mcl-PHA. Scl-PHA are thermoplastic materials, while mcl-PHA tend to possess physical characteristics that are similar to those of rubbers.

Varying the nature of the carbon source that is used in the bacterial culture, a wealth of different side-chain structures may be obtained, giving rise to diverse biomaterials that are used for various applications, particularly medical applications. For instance, scl-PHA are considered to be suitable for hard tissue engineering or bone replacement material, while mcl-PHA are more attractive for applications such as heart valves, vascular grafts, skin tissue engineering, wound healing, and controlled drug delivery [10,11,12].

PHA have found applications in the vast fields of nanotechnology due to their compatibility and uniform chirality and are used as starting chemicals for many other end products [13]. It is also possible to introduce certain functional groups in the side chain, including double bonds, which may allow for further reactions, such as the crosslinking of the polymer chains [14].

Depending on the degree of unsaturation, mcl-PHAs have a number of potential applications, including biodegradable elastomers and adhesives. When provided with a substrate that is high in unsaturated fatty acids, such as soybean oil, a mcl-PHA results, with a high concentration of side-chain olefinic group [15,16].

Olefinic groups can also be chemically modified to produce polymers with different properties [17].

**Figure 2:**
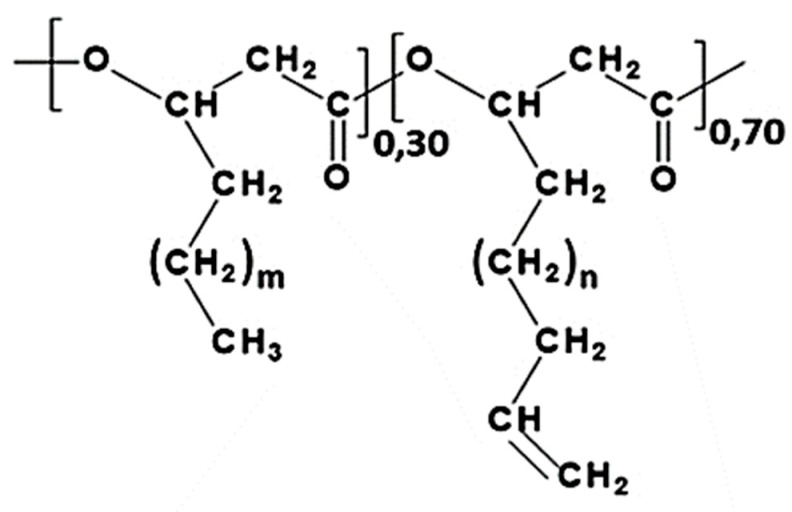
Chemical structure of the PHA synthesized by *P. mediterranea* cultured on a medium containing sodium octanoate and 10-undecenoic acid. The m index may take the values 1, 3, and 5, the n index the values 0, 2, and 4.

The aim of this study was the evaluation of a PHA that is synthesized by *Pseudomonas mediterranea*, and cultured onto a medium containing a mixed carbon source of sodium octanoate and 10-undecenoic acid [14], as a material to prepare nanoparticles that are potentially useful as drug carriers. This polymer, labelled as Uns-PHA because of its unsaturation, is characterized by the presence of terminal double bonds in the structure of the side chains, as shown in Figure 2. This feature makes it particularly sensitive to physical and chemical radical initiators, leading to the formation of a cross-linked network.

Nanoparticles were produced from the PHA by a nanoprecipitation method, with the goal of reaching the technological features that were particularly suitable for ocular formulations, such as a mean hydrodynamic radius of below 200 nm. The affinity of the tested polymer towards the hydrophilic and lipophilic model compounds, the stability over time under different storage conditions, and the possibility of freeze-drying the nanoparticle suspensions were also investigated, in view of their pharmaceutical applications. 

Besides, this study was also focused on the conditions at which the cross-linking process of the tested polymer can occur, as well as the effects of cross-linking on the technological features of the resulting nanoparticles, compared to those produced from the starting polymer. In particular, the role of physical radical initiators was investigated, to understand in which conditions the original polymer or preformed nanoparticles underwent chemical events (i.e., the formation of inter/intramolecular bonds) that would irreversibly change their physico-chemical properties.

## 2. Results and Discussion

### 2.1. Physico-Chemical Characterization of Nanoparticles

**Figure 3:**
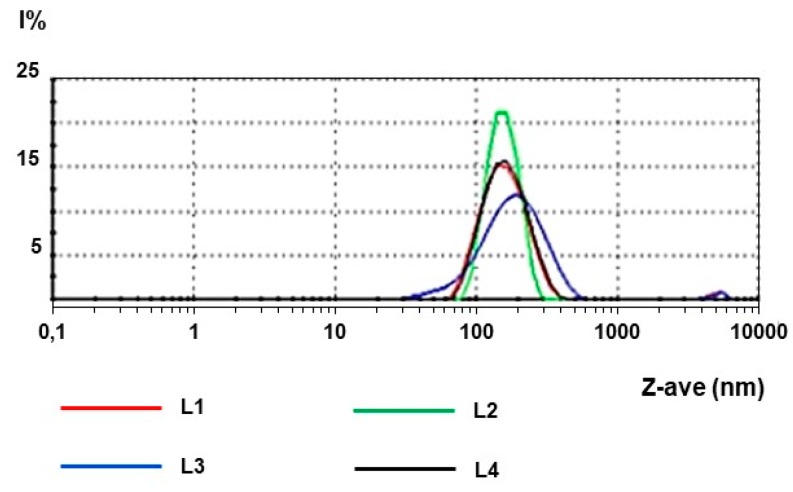
Dimensional distribution of the four batches of unloaded nanoparticles.

Uns-PHA was synthesized, purified, and structurally characterized as described in the “Materials and Methods” section (Paragraphs 3.1 and 3.2). The physico-chemical characterization confirmed that the polymer Uns-PHA is able to form discrete nanoparticles with a mean size that is compatible with the ophthalmic administration (Figure 3). The mean particle size (Z-Ave) value was 154.8 nm, and the polydispersity index (PdI) was lower than 0.4, suggesting the presence of highly homogeneous systems. The surface net charge of these nanoparticles (ZP) was negative. The experimental data shown in Table 1 endorsed the reproducibility of the method used for nanoparticle production. 

Nanoparticles made from polymer Uns-PHA-incorporated calcein with a high efficiency (EE% = 75.57), and this did not affect their mean diameter (Table 2). Also in this case, the PdI value was low and ZP was negative, which were comparable with the values of the unloaded nanoparticles. Therefore, calcein seems to have been incorporated homogeneously within the polymer matrix, producing no variation in the physico-chemical parameters of the nanoparticles.

Analogously, with Nile red the Z-Ave remained almost unchanged compared to the unloaded nanosystems, the PdI values were lower than 0.3, and the ZP remained almost unchanged (Table 3). Therefore, it is possible to suppose also for this lipophilic probe, which was loaded with a very high efficiency (EE% = 83.6), that homogeneous incorporation occurred within the polymeric matrix.

### 2.2. Stability Studies

**Figure 4:**
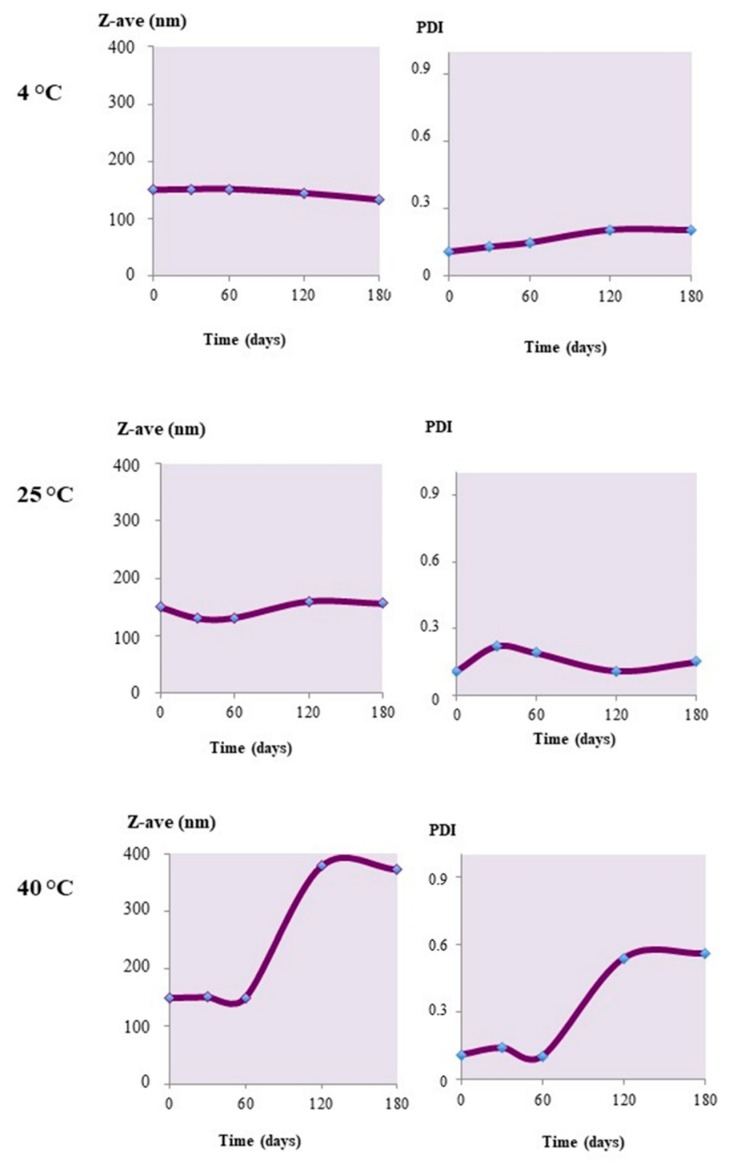
Z-Ave and PdI changes upon the storage of unloaded Uns-PHA nanoparticles at different temperatures.

The unloaded nanoparticles showed a good degree of physical stability under different storage conditions. The Z-Ave values did not change over time, remaining almost constant for up to four months when stored at 4 °C or at 25 °C (Figure 4). In the latter case, some small changes were registered in the nanoparticle sizes. Also, the PdI value, under the same conditions, remained unchanged, with values below 0.4, indicating a certain stability of the colloidal suspensions. When kept at 40 °C instead, a size increase was observed, which was associated with an increase in the PdI. This behavior would indicate the occurrence of aggregation phenomena at higher storage temperatures.

**Figure 5:**
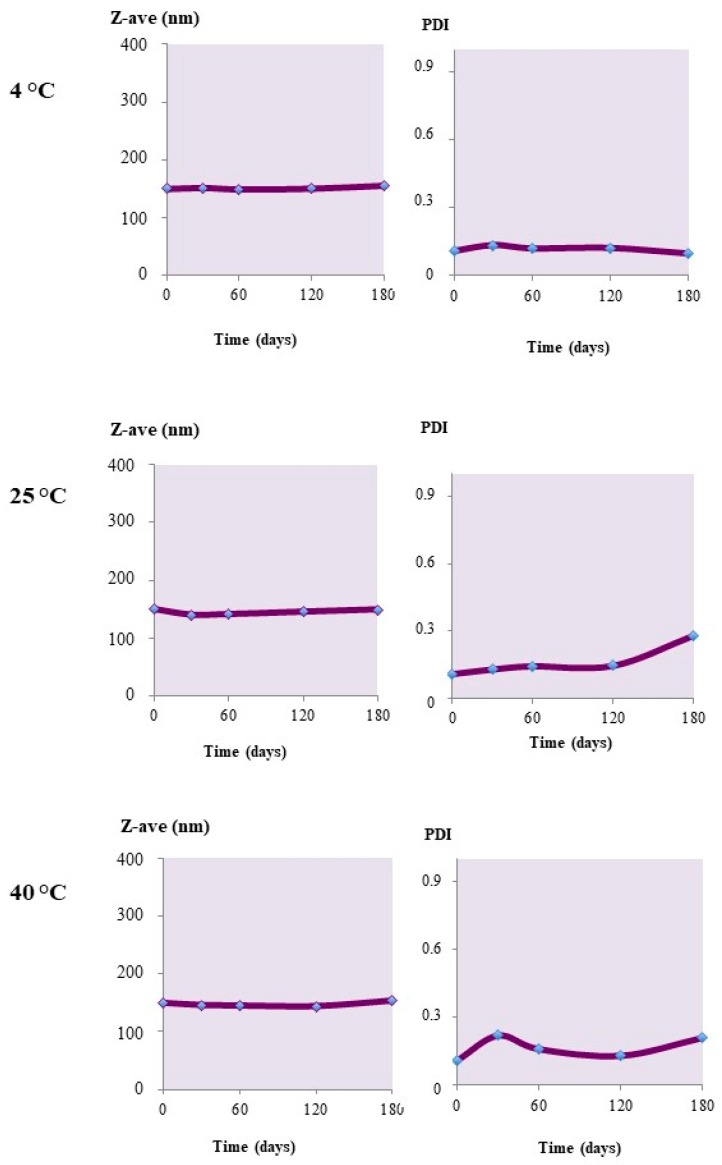
Z-Ave and PdI changes of calcein-loaded Uns-PHA nanoparticles stored at different temperatures.

In the case of the nanoparticles loaded with calcein or Nile red, analogous behavior was registered. Formulations that were prepared with the hydrophilic probe calcein were stable in the different storage conditions applied. Also, during storage at 40 °C (Figure 5), the mean particle diameter and PdI remained almost unaltered. This behavior contrasts with the stability patterns of the unloaded carriers, which instead seemed to be sensitive, even at high temperatures (Figure 4). 

**Figure 6:**
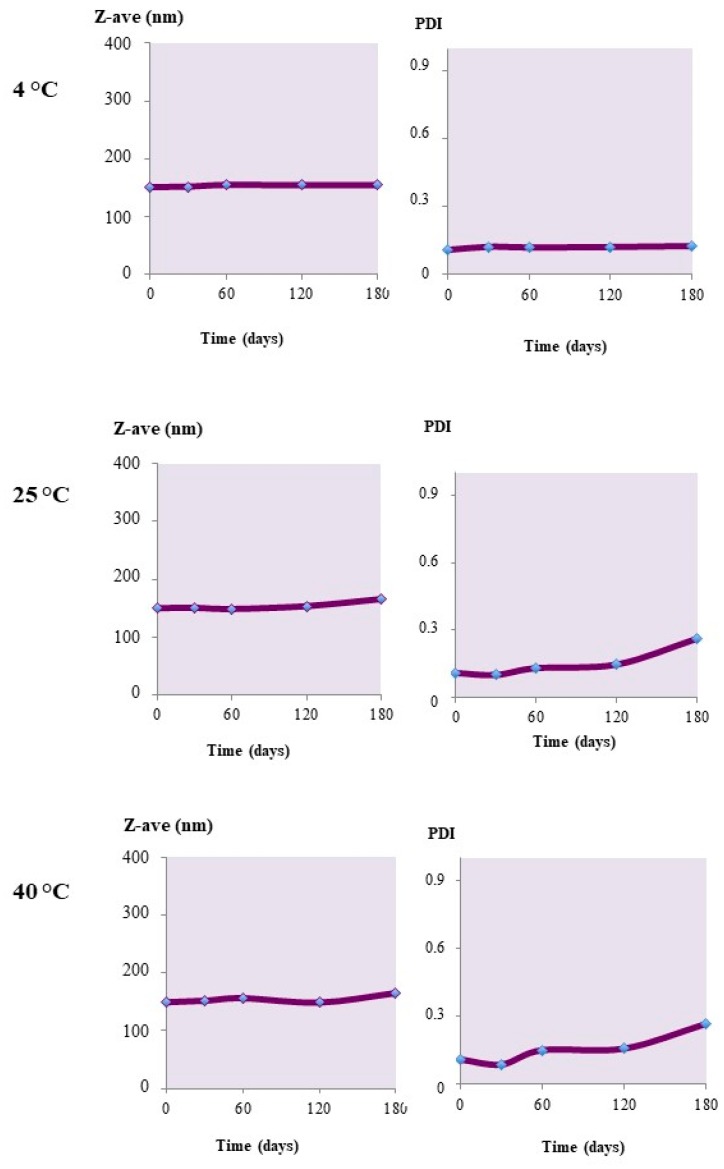
Z-Ave and PdI changes of Nile red-loaded Uns-PHA nanoparticles stored at different temperatures.

Also, the nanoparticles loaded with Nile red, as shown in Figure 6, were highly stable at all of the adopted storage conditions. Since in these experiments, the stability was settled in terms of the maintenance of the original nanoparticle mean size, it is conceivable that in the presence of the loaded cargoes, the tendency to aggregate was reduced with respect to the unloaded systems, and regardless of the surface charge (Zeta potential), which was measured to be almost equivalent between the three series of nanoparticles.

### 2.3. Influence of Cryoprotectants in the Freeze-Drying of Nanoparticles

Table 4 shows the results of the studies carried out for the evaluation of the most suitable cryoprotectant for the lyophilization of unloaded Uns-PHA nanoparticles.

Only the results for Hydroxypropyl-β-cyclodextrin (HP-β-CD) at a concentration of 1% (*w*/*v*) showed that the nanosuspension maintained its initial size. With the other tested auxiliaries, conversely, an apparent absence of protection against the agglomeration caused by the freezing process was registered. 

### 2.4. Induction of Cross-Linking

^1^H-NMR characterization allowed to establish the structure of Uns-PHA (Figure S1). Due to a double terminal bond on the lateral chain, we investigated the tendency for this polymer to form intra/intermolecular bridges, modifying its physico-chemical structure. Possible crosslinking reactions occurring between the unsaturated bonds in the polymer are oxidative reactions in the presence of water or oxygen, and/or dimerizations giving rise to cyclobutane rings [18]. This last reaction is illustrated in Figure S2. Other evidence is reported in the literature on the crosslinking of unsaturated PHAs upon exposure to UV irradiation [16,19,20], where crosslinking by the free radical mechanism was ascertained by comparing ^1^H-NMR, Infrared spectroscopy (IR), Gas Chromatography-Mass Spectrometry (GC-MS), and sol-gel analysis of opportunely treated vs. untreated specimens. 

Moreover, we hypothesized that the aggregation of Uns-PHA into nanoparticles could result in a change in reactivity towards the cross-linking-inducing physical stimuli. Cross-linked polymeric nanoparticles, as well as nanoparticles made using a cross-linked polymer, are expected to act differently in terms of drug loading, drug release, physical stability, and biodegradability, compared to those that are produced by using the native, non-cross-linked material. These kinds of comparative studies have been planned in a forthcoming work. 

### 2.5. Incandescence Lamp

Based on previous experiments from our lab (data not shown), cross-linking was induced by UV irradiation at a wavelength of 365 nm, at room temperature and at atmospheric pressure, but varying by the length of the experiment. 

**Figure 7:**
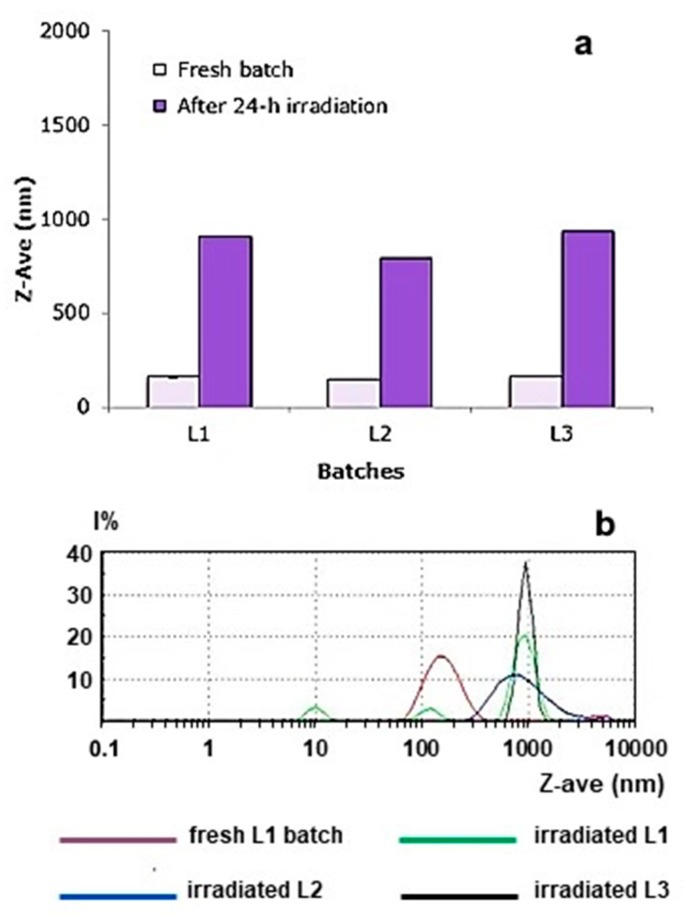
(**a**) Changes of Z-Ave values of blank nanoparticles after 24-h exposure to an incandescent lamp; (**b**) experimental results of the photon correlation spectroscopy (PCS) analysis for batches L1, L2, and L3 after exposure to light, compared to the fresh L1 batch.

To verify whether the exposure of nanoparticles for 24 h to an incandescent yellow lamp induced the cross-linking reaction, a size analysis was made in comparison to the native batches. Figure 7 shows that the mean particle diameter of all the three tested batches increased considerably. This would confirm that the samples were physically altered by the application of yellow light.

The second test, carried out to determine whether an alteration of the polymer structure had taken place, was the observation of any difference of solubility in acetone, a solvent in which the untreated nanoparticles and the Uns-PHA films were easily soluble. The results of turbidimetric analysis (at λ = 550 nm) showed a loss of solubility of the treated samples in acetone (Table 5), confirming substantial changes in the characteristics of the polymer, most probably as a consequence of its cross-linking. 

### 2.6. UV Irradiation at 300 nm

**Figure 8:**
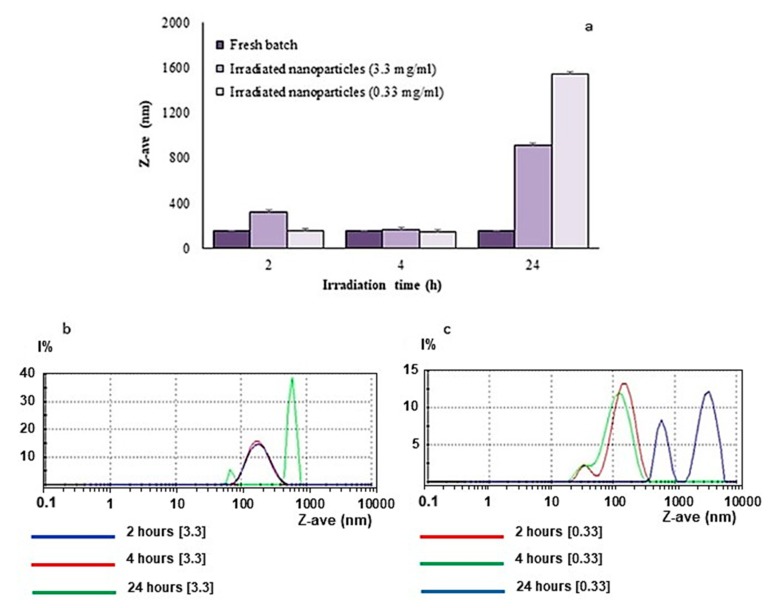
(**a**) Changes in the mean particle sizes of the nanoparticle suspensions at two different concentrations irradiated at 300 nm for various times; (**b**) size distribution after 4 h UV-lamp irradiation of nanoparticles at a concentration of 3.3 mg/mL; (**c**) size distribution after 24-h UV-lamp irradiation of nanoparticles at a concentration of 0.33 mg/mL.

**Figure 9:**
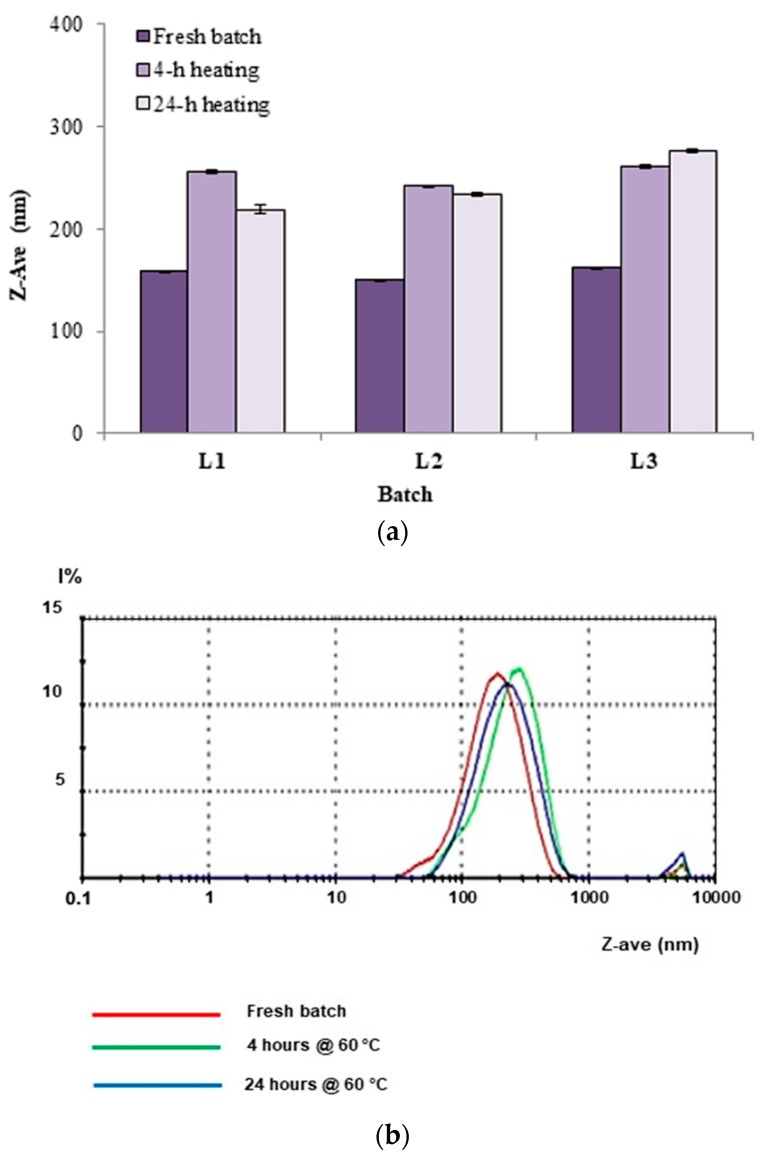
(**a**) Z-Ave values of fresh nanoparticle suspensions or after heating at 60 °C for 4 or 24 h; (**b**) size distribution of nanoparticles kept in at 60° C for 4 or 24 h.

Figure 8 shows that UV irradiation for 2 h and 4 h of unloaded nanoparticle suspension, at a concentration equal to 3.3 mg/mL, did not produce a significant variation in size distribution. Conversely, after 24 h of irradiation, the average particle size increased considerably; this suggests that the nanoparticle suspension changed its properties, and that cross-linking may have occurred. Similar results were registered when unloaded nanoparticles were irradiated at a concentration of 0.33 mg/mL (Figure 9). In this case, after a 24 h irradiation period, the appearance of multiple dimensional peaks was observed, with a diameter in the order of thousands of micrometers, indicating that a long irradiation time induced aggregative phenomena. Such an effect appeared to be more pronounced in this diluted specimen than in the more concentrated one (above), probably as a consequence of an easier penetration of the UV rays in the exposed sample.

### 2.7. Heating

Induction of cross-linking through the use of heat (60 °C) did not produce any significant changes in the size distribution of the tested nanoparticle batches (Figure 9). In this case, the result is not unexpected because, in the heating experiments previously reported [16], benzoyl peroxide was used as a radical initiator.

## 3. Materials and Methods

Poly(oxyethylensorbitanmonooleate) (Tween^®^ 80), phosphate-buffered saline (PBS) tablets (pH 7.4), undecylenic acid (≥96%), sodium octanoate (≥99%) and solvents were all purchased from Sigma-Aldrich srl (Milan, Italy). HPLC-grade water was used throughout the work. 

### 3.1. Biosynthesis of Poly(3-Hydroxyalkanoate) (Uns-PHA)

*Pseudomonas mediterranea* IPVCT 9.1, kindly provided by the Institute of Plant Pathology, University of Catania, Catania, Italy, was cultivated in Luria Bertani (LB) broth at 30° C under continuous shaking (250 rpm). The strain was stored in 20% glycerol at −80 °C, and maintained on LB agar plates for routine use.

Uns-PHA biosynthesis was carried out in E* medium containing the following (per liter): 0.55 g (NH_4_)_2_HPO_4_, 5.8 g K_2_HPO_4_, 3.7 g KH_2_PO_4_, 10 mL 0.1 M MgSO_4_, supplemented with 1 mL of a microelement solution (MT solution). The MT solution contained the following salts (per liter): 2.78 g FeSO_4_∙7H_2_O, 1.98 g MnCl_2_∙4H_2_O, 2.81 g CoSO_4_∙7H_2_O, 1.67 g CaCl_2_∙2H_2_O, 0.17 g CuCl_2_∙2H_2_O, 0.29 g ZnSO_4_∙7H_2_O. Undecylenic acid and sodium octanoate were added at final carbon concentration of 10 mM (1.84 g/L) and 15 mM (2.49 g/L), respectively. The medium was adjusted to pH 7.0 and sterilized in autoclave at 121 °C for 20 min. *P. mediterranea* cells, precultured in LB broth, were harvested by centrifugation (5000× *g* for 10 min at 25 °C), washed three times with sterile E* medium, resuspended in 100 mL up to an OD_540_ = 0.3 (corresponding to 3 × 10^8^ cells∙mL^−1^) and inoculated in 1 l of E* medium. Fermentation was performed in a shaker at 250 rpm under aerobic conditions at 30 °C. After 96 h, the cells were collected by centrifugation (5000× *g* for 10 min at 25 °C), washed twice in PBS, and lyophilized.

### 3.2. PHA Extraction

The polymer was isolated by chloroform extraction of the lyophilized cells in a Soxhlet apparatus. After refluxing for 6 h, the chloroform was removed with a rotavapor. The residue was dissolved in chloroform (1:5 *w*/*v*), and precipitated into 10 parts of rapidly stirred ethanol. Stirring was continued for 30 min, after which the solvent mixture was left to stand overnight. The collected material was separated by centrifugation (Beckman J2-21, JA-20 rotor, Palo Alto, CA, USA; 20 °C; 9000× *g*), washed twice with ethanol, and dried at reduced pressure (1 mm Hg) at room temperature. This purification step was repeated to obtain the final polymer.

The resulting polymer was characterized by means of ^1^H-NMR analysis (see Supplementary Materials) with an Agilent INOVA instrument (Agilent Technologies, Santa Clara, CA, USA) operating at 500 MHz.

### 3.3. Preparation of Nanoparticles

An organic phase, made by dissolving the polymer in acetone at a concentration of 0.66 mg/mL was slowly dropped by a Pasteur pipette into a solution of Tween 80 in water (0.5%, *w*/*v*), under constant magnetic stirring at 600 rpm at room temperature. The organic/water phase volume ratio was 1:2. To assess the capacity of the polymer to encapsulate drugs, a model hydrophilic probe (calcein) and a lipophilic one (Nile red) were used; calcein was added at a 2% (*w*/*v*) concentration in the aqueous phase, whereas Nile red was dissolved at the same concentration in the acetone solution. The solvent was finally evaporated off under a rotating vacuum at 40 °C.

### 3.4. Physico-Chemical Characterization of Nanoparticles

The prepared SLN batches were subjected to PCS analysis, using a Nanosizer ZS90 (Malvern Panalytical Ltd, Malvern, UK) connected to a PC running the dedicated PCS v1.27 software from the same company. To measure the mean size (Z-ave) and polydispersity index (PdI), an aliquot of each sample was diluted 10-fold with HPLC-grade water, and placed in a glass cuvette; measurements were done by a laser beam at a wavelength of 633 nm. The reported values are the mean ± SD of 90 measurements (three sets of 10 measurements in triplicate). The zeta potential (ZP) was determined by electrophoretic light scattering with the same instrument. Up to 100 measurements on each sample were registered at room temperature, to calculate the electrophoretic mobility, and using the Smoluchowski constant (Ka) with a value of 1.5, the corresponding ZP values.

### 3.5. Determination of the Encapsulation Efficiency

A known volume of each nanoparticle suspension was centrifuged at 13,000 rpm and 4 °C for 30 min (IEC CENTRA MP4R, Waltham, MA, USA). In the case of Nile red, the resulting pellet was dissolved with acetonitrile under vortex-mixing and the solution was read by an UV spectrophotometer (Shimadzu UV-1601, Shimadzu Italia, Milan, Italy) at λ_max_ = 536 nm, using a calibration curve of Nile red in the same solvent that showed linearity (r^2^ = 0.9989) in the range 0.5–10 µg/mL. For calcein, the pellet was destroyed with acetone, the solution was dried off under a nitrogen flow and the probe was redissolved with water. After filtration (0.45 µm nylon filter membrane) the solution of calcein was read by UV spectrophotometry at 491 nm, using a calibration curve of calcein in water that was linear in the range 2.5–50 µg/mL (r^2^ = 0.9956). The encapsulation efficiency (EE%) for each sample was calculated as:

EE% = Ce/Co × 100

where Ce and Co are the amount of encapsulated probe and the initially added amount of probe respectively present in one mL of nanoparticle suspension. 

### 3.6. Stability Tests

An aliquot of each formulation was stored in closed Eppendorf vials at room temperature (at 25 ± 2 °C), in a refrigerator (at 4 ± 1 °C), or in an oven (at 37 ± 2 °C). Z-ave and PdI values were determined every 30 days for six months, by diluting each sample 10-fold with water before the PCS measurement.

### 3.7. Studies on Freeze-Drying and Cryoprotectants

The nanoparticle suspensions (2 mL) were added with the cryoprotectant listed in Table 4, accurately mixed, and then frozen under liquid nitrogen. After 24 h lyophilization (Edwards Modulyo) the samples were reconstituted with 2 mL water and submitted for PCS analysis. 

### 3.8. Cross-Linking Studies

#### 3.8.1. Cross-Linking Induction by a Yellow Light Incandescent Lamp

Three batches of unloaded nanoparticles (L1, L2, and L3), at a polymer concentration of 3.3 mg/mL were selected. Two milliliters of each sample was placed for 24 h in front of a yellow light incandescent lamp, at a distance of 15 cm. Analogously, a solution of Uns-PHA in acetone (3.3 mg/mL) was evaporated off under a nitrogen flow in a rotating glass tube to obtain a thin film, which was placed under the same irradiation conditions above.

After the treatment, the nanoparticles were centrifuged (13,000 rpm at 4 °C for 30 min), and the pellet was treated with 10 mL acetone; similarly, the polymer film after irradiation was treated at room temperature with 10 mL acetone. Any incomplete dissolution was checked out by turbidimetric analysis, as the appearance of an UV signal at λ = 550 nm. 

#### 3.8.2. Cross-Linking Induction by UV Irradiation

To assess the effect of polymer concentration on the cross-linking process, two nanoparticle specimens were prepared by diluting the initial suspension so that to achieve a polymer concentration of 3.3 and 0.33 mg/mL, respectively. Also in this experiment, a corresponding polymer film was produced, as previously described. All samples were exposed to 12 UV lamps at λ = 300 nm for a total 24 of h. Aliquots from each sample were withdrawn after 2, 4, and 24 h of exposition, to assess by PCS analysis any size change (after 1:10 dilution with water). 

#### 3.8.3. Cross-Linking Induction by Heating

The same L1, L2, and L3 batches (3.3 mg/mL of polymer) were placed in a Büchi mini-oven at 60 °C. Aliquots were withdrawn after 4 and 24 h of incubation and analyzed by PCS.

## 4. Conclusions

This study demonstrated that the unsaturated polyester Uns-PHA is able to form nanoparticles that meet the required physico-chemical and technological parameters for a potential ophthalmic application, mainly in terms of particle size (the experimental average value was 150 nm), surface charge (Zeta potential), stability in physiological means, and the possibility for producing freeze-dried materials that could be stored for longer times. 

In particular, stability studies at 4 or 25 °C gave very positive information for both the unloaded nanoparticles, and for those being incorporated with the two probes. Freeze-drying tests demonstrated that nanoparticles can be lyophilized and resuspended in an aqueous medium, without compromising their original size distributions, especially when using HP-β-CD at 1% (*w*/*v*) as a cryoprotection agent. 

Cross-linking tests that were carried out using physical radical initiators showed that this polymer tends to cross-link both nanoparticles under the action of UV rays, and after irradiation with an incandescent yellow lamp. The use of temperature as a promoter of cross-linking, instead did not succeed. Apart from the form of a thin polymeric film, even when it was structured as nanoparticles, the polymer was reactive to the physical stimuli, and it underwent cross-linking.

All of these experimental data would suggest the potential use of Uns-PHA, a polyester of microbial origin, in the field of controlled drug delivery and nanomedicine. Due to the small particle size obtained, with the consequent possibility of easily sterilizing the nanosuspension by membrane filtration, its potential use in the ophthalmic area can be positively anticipated.

## Figures and Tables

**Table 1:** **Mean particle size** (Z-Ave), polydispersity index (PdI), and Zeta potential (ZP) values for unloaded nanoparticles.

Batch	Z-Ave (nm)	PdI	ZP (mV)
L1	158.1	0.150	−17.8
L2	149.7	0.064	−24.8
L3	162.0	0.235	−20.0
L4	149.5	0.109	−22.8
Mean	154.8	0.140	−21.4
± S.D.	6.21	0.10	3.13

**Table 2:** Experimental data for calcein-loaded nanoparticles.

Batch	Z-Ave (nm)	PdI	ZP (mV)	EE%
L1c	154.8	0.213	−18.9	78.6
L2c	148.2	0.067	−26.1	69.9
L3c	152.5	0.083	−22.1	77.7
L4c	181.1	0.253	−22.2	80.1
Mean	159.2	0.154	−22.3	76.57
± S.D.	14.91	0.12	2.92	4.58

**Table 3:** Experimental data for the Nile red-loaded nanoparticles.

Batch	Z-Ave (nm)	PdI	ZP (mV)	EE%
L1n	143.2	0.101	−22.1	83.4
L2n	157.6	0.170	−25.8	71.9
L3n	147.6	0.276	−23.1	90.2
L4n	155.1	0.201	−24.8	88.9
Mean	150.9	0.187	−24.0	83.6
± S.D.	6.62	0.13	1.62	8.34

**Table 4:** Results of the freeze-drying and cryoprotection studies. The tested nanoparticle batches had a mean particle size of 158.1 nm before lyophilization.

Cryoprotectant	Concn.	Z-Ave (nm)	PdI
	(% *w*/*v*)	(post-lyophilization)	
Trehalose	5	694.9	0.878
Trehalose	10	558.6	0.531
Lactose	10	623.2	0.479
Glucose	10	323.1	0.406
Hydroxypropyl-β-cyclodextrin (HP-β-CD)	1	191.4	0.184

**Table 5:** Solubility of Uns-PHA nanoparticles in acetone, along with a corresponding thin film, before and after exposure to an incandescent yellow lamp. Two milliliters of nanoparticle samples (containing 6.6 mg of polymer) or the same amount of polymeric film were treated with 10 mL acetone; any lack of solubility was determined by a qualitative assay, registering the presence of turbidity (UV analysis at 500 nm) in native or irradiated samples.

Nanoparticle Batch	Before	After
L1	Soluble	Insoluble
L2	Soluble	Insoluble
L3	Soluble	Insoluble
L4	Soluble	Insoluble
Polymer film	Soluble	Insoluble

## Supplementary Materials

The following are available online at https://www.mdpi.com/1996-1944/12/6/868/s1, Figure S1: The chemical structure of the PHA used in this study, and its 500 MHz ^1^H NMR spectrum in CDCl_3_, with assignments. The m index may take the values 1, 3, and 5, and the n index, 0, 2, and 4. The singlet peak with an asterisk at 2.17 ppm is due to acetone. Figure S2: Schematic illustration of the cross-linking process of uns-PHA under the effect of light.
