# Supplementary material for: Unsaturated Poly(Hydroxyalkanoates) for the Production of Nanoparticles and the Effect of Cross-Linking on Nanoparticle Features
Source: Materials (Basel). 2019 Mar 15;12(6):868. doi: 10.3390/ma12060868 (PMC6471160; doi:10.3390/ma12060868)
Supplement: Supplementary file 1 [file materials-12-00868-s001.pdf]

materials

MDPI

Supplementary Materials: Unsaturated Poly(hydroxyalkanoates) (PHA) for the Production of Nanoparticles and Effect of Cross-Linking on Nanoparticle Features.

Rosario Pignatello, Giuseppe Impallomeni, Sarha Cupri, Giuseppe Puzzo, Claudia Curcio, Maria Giovanna Rizzo, Salvatore Guglielmino and Alberto Ballistreri

1. H-NMR characterization of P_{279} polymer

The ^{1}H-NMR spectrum of the PHA used in this study is given in Fig. S1, together with its structure and the signal assignment.

The olefinic protons 8 and 9 resonate at 5.77 and 4.98 ppm, and have the correct intensity ratio of 1:2. The main chain protons 3 and 2 signals are found at the characteristic PHA chemical shift of 5.18 and 2.53 ppm. Also this couple of signals has the expected intensity ratio of 1:2. The peak of the terminal methyl group of saturated units is present at 0.88 ppm. The area of this peak and those of the signals of the olefinic protons may be used to calculate the percentage of unsaturated units in the polymer (% Uns) with the following formula:

$$\% \text{ Uns} = (I_{5.77} + I_{4.98})/(I_{5.77} + I_{4.98} + I_{0.88}) \quad (1)$$

where $I_{5.77}$, $I_{4.98}$, and $I_{0.88}$ are the integrated areas of the peaks at the chemical shift indicated by the subscript number. The % Uns results 70%.

The remaining assignments were confirmed by a COSY spectrum (data not shown). The % Uns may be calculated also from the intensity of peak 7 at 2.05 ppm due to the methylene protons next to the double bond with the following formula:

$$\% \text{ Uns} = \tfrac{1}{2} I_{2.05} / (\tfrac{1}{2} I_{2.05} + \tfrac{1}{3} I_{0.88}) \quad (2)$$

yielding the same result of 70%.

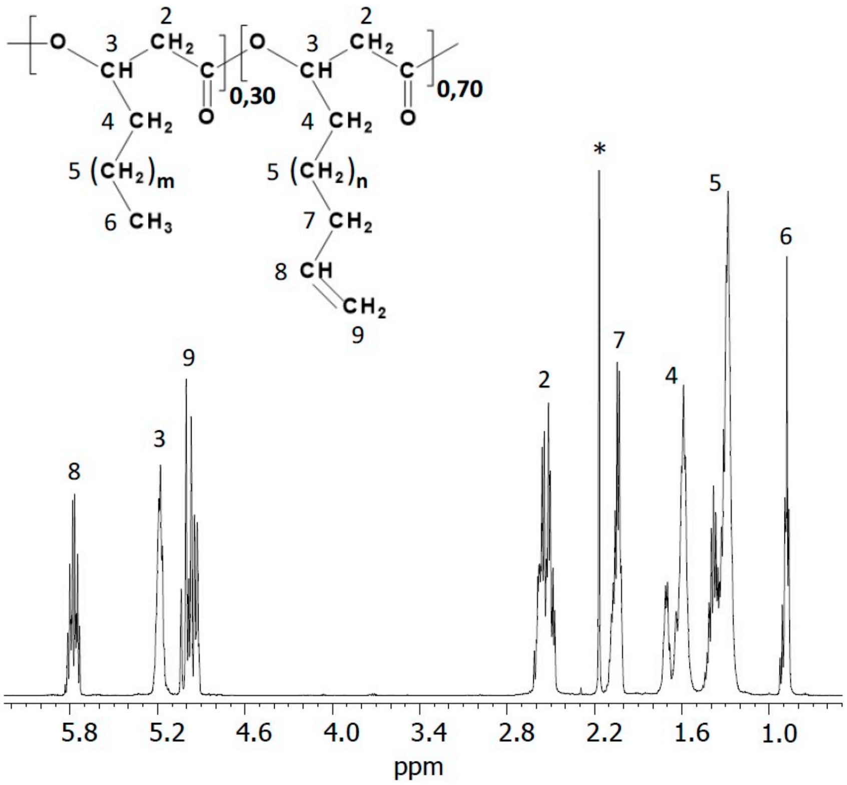

Figure S1. Chemical structure of the PHA used in this study and its 500 MHz ^1H NMR spectrum in $CDCl_3$, with assignments. The m index may take the value 1, 3, and 5, the n index 0, 2, and 4. The singlet peak with an asterisk at 2.17 ppm is due to acetone.

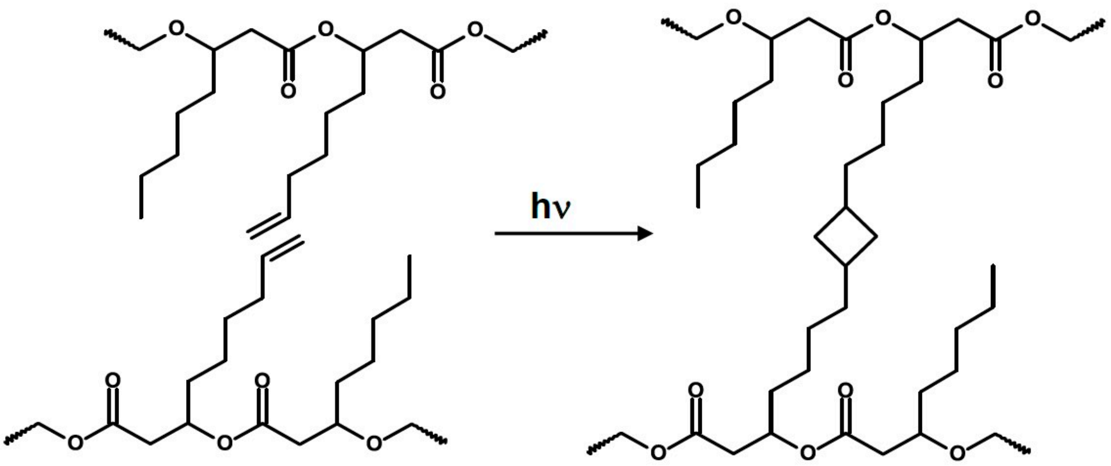

Figure S2. Schematic illustration of the cross-linking process of uns-PHA under the effect of light.

© 2019 by the authors. Submitted for possible open access publication under the terms and conditions of the Creative Commons Attribution (CC BY) license (http://creativecommons.org/licenses/by/4.0/).
